# Guaiacol Nitration in a Simulated Atmospheric Aerosol with an Emphasis on Atmospheric Nitrophenol Formation Mechanisms

**DOI:** 10.1021/acsearthspacechem.1c00014

**Published:** 2021-04-12

**Authors:** Ana Kroflič, Janine Anders, Ivana Drventić, Peter Mettke, Olaf Böge, Anke Mutzel, Jörg Kleffmann, Hartmut Herrmann

**Affiliations:** † Department of Analytical Chemistry, National Institute of Chemistry, Hajdrihova 19, 1000 Ljubljana, Slovenia; ‡ Atmospheric Chemistry Department (ACD), Leibniz-Institute for Tropospheric Research (TROPOS), Permoserstraße 15, 04318 Leipzig, Germany; § Physical and Theoretical Chemistry, University of Wuppertal, Gaußstraße 20, 42119 Wuppertal, Germany

**Keywords:** guaiacol, nitrophenol formation, aerosol chamber, HONO, dark nitration, carbon loss, catechol, aqueous-phase nitration

## Abstract

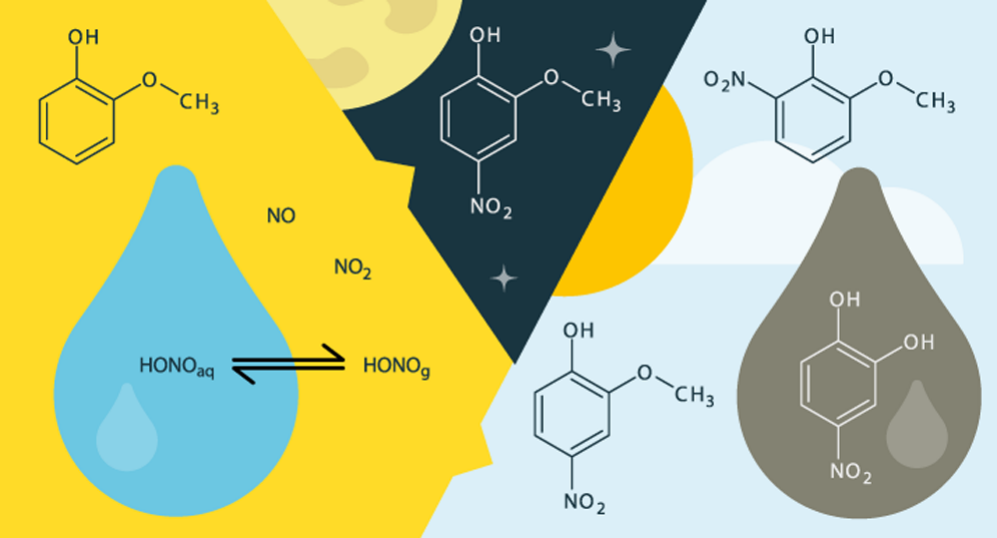

Atmospheric nitrophenols are pollutants of concern due to their toxicity and light-absorption characteristics and their low reactivity resulting in relatively long residence times in the environment. We investigate multiphase nitrophenol formation from guaiacol in a simulated atmospheric aerosol and support observations with the corresponding chemical mechanisms. The maximal secondary organic aerosol (SOA) yield (42%) is obtained under illumination at 80% relative humidity. Among the identified nitrophenols, 4-nitrocatechol (3.6% yield) is the prevailing species in the particulate phase. The results point to the role of water in catechol and further 4-nitrocatechol formation from guaiacol. In addition, a new pathway of dark nitrophenol formation is suggested, which prevailed in dry air and roughly yielded 1% nitroguaiacols. Furthermore, the proposed mechanism possibly leads to oligomer formation via a phenoxy radical formation by oxidation with HONO.

## Introduction

Biomass burning (BB) is an important source of atmospheric organic pollutants, while its contribution to poor air quality is believed to still increase with global warming due to the increased incidence of natural fires in arid conditions.^1^ Among the most important properties of primary BB emissions is the potential to form light-absorbing organic aerosol particles also termed brown carbon (BrC), which, besides their light-absorbing characteristics, influence the oxidative capacity of the atmosphere and induce climate change.^2−4^ Although BrC is highly variable in sources and identity, its absorptive characteristics have often been found importantly altered by a small fraction of nitrated phenols (NPs) connected with BB sources.^5−9^ However, the secondary formation of NPs from their semivolatile aromatic precursors that often result from the breakdown of lignin during biomass burning is very poorly understood.^10−12^

Daytime reactions of differently substituted phenols with OH radicals in the presence of NO*_x_* are generally considered a dominant pathway of ambient NP formation, followed by the nighttime NO_3_-mediated chemistry.^10^ This, however, is counterintuitive to observed diurnal profiles from the field, which often exhibit maximal NP concentrations at night.^5,9,10,12^ Different scientists tend to explain ambient observations differently, by rapid daytime chemistry of atmospheric NP (direct photolysis or induced by reactions with OH radicals)^10,13−15^ and/or by additional nighttime sources. The latter include gas-phase reactions with NO_3_,^16^ heterogeneous reactions of particulate methoxyphenols with NO_2_ or NO_3_ radicals^17,18^ and different aqueous-phase mechanisms that have recently been studied in the laboratory environment.^19−26^

Among the most studied model substances of BB emissions is 2-methoxyphenol (guaiacol, GUA), a volatile organic compound that originates from wood lignin and predominantly resides in the atmospheric gaseous phase. As a result of atmospheric processing, nitrated guaiacol (NG) species constitute secondary organic aerosols (SOAs) and contribute to the atmospheric absorption in the near-UV and visible ranges.^27,28^ NGs, however, are a minor fraction of aged SOA mass formed from GUA; organic acids and oligomer formation have been shown to be predominant under different NO*_x_* and humidity conditions.^29−31^ Moreover, laboratory SOA produced in those studies always resembled typical O/C ratios of aged atmospheric particles (O/C ∼ 1). Although a few laboratory and chamber studies exist on SOA formation from GUA by reactions with hydroxyl radicals (OH), ozone (O_3_), and organic triplet excited states (^3^C*),^29−32^ aromatic nitration has not been in focus in any of them.

Within the present study, we put emphasis on the formation of colored NPs during GUA aging in a polluted NO*_x_* environment affected by intensive BB, which was investigated by chamber experiments. The multiphase study design was such that it allowed for the validation of recently proposed HONO-assisted aqueous-phase mechanisms for the nitration of BB phenols in the atmosphere.^24^ Therefore, the applied conditions were not typical for dark atmospheric processing because the formation of the most important NO_3_ radicals was prevented. We rather focused on a mixture of NO/NO_2_/HONO, which under illumination also produces other typical daytime atmospheric oxidants (e.g., OH radicals). Established nitration mechanisms were reviewed and discussed in light of our observations. Dark nitration and the role of water were specifically addressed, which brought up a new nitration pathway in the dark, involving HONO.

## Experimental Section

### Chemicals

Nine commercially available standard compounds were used for quantification purposes: 2-methoxyphenol (guaiacol, GUA), pyrocatechol (catechol, CAT), pyrogallol (GAL), 4-nitroguaiacol (4NG), 2-methoxy-5-nitrophenol (5-nitroguaiacol, 5NG), 2-methoxy-6-nitrophenol (6-nitroguaiacol, 6NG, Key Organics/BIONET, U.K.), 6-methoxy-2,4-dinitrophenol (4,6-dinitroguaiacol, (4,6DNG), AKos GmbH, Germany), 4-nitrocatechol (4NC), and 3,5-dinitrocatechol (DNC). Purity of all standards was ≥95%. GUA was also used as a precursor compound in the aerosol chamber experiments. Additionally, nitropyrogallol was synthesized in an aqueous-phase photoreactor (for details see Supporting Information (SI)). Liquid chromatography (LC) and liquid chromatography–mass spectrometry (LC–MS) grade solvents and ultrapure water supplied by a Millipore Milli-Q purification system were used for solution preparation, extraction, and mobile-phase preparation.

### Experimental Setup: ACD-C Simulation Chamber

Experiments were conducted in a cylindrical Teflon aerosol chamber with a volume of 19 m^3^. The chamber is placed inside of a thermostated construction, equipped with visible light sources (16 bulbs were used to obtain actinic fluxes in the UV–Vis simulating solar irradiation at *J*(NO_2_) = 2.6 × 10^–3^ s^–1^) and allows for conditions of up to 80% relative humidity (RH), which is necessary for studies of multiphase chemical processes in deliquesced particles. The chamber is commonly equipped with multiple online instruments, such as a scanning mobility particle sizer (SMPS), a UV photometric O_3_ analyzer (model 49*i*, Thermo Scientific), and a proton transfer reaction (time-of-flight) mass spectrometer (PTR-(TOF)MS, Ionicon). Besides, we also used a long path absorption photometer (LOPAP) to measure gaseous nitrous acid (HONO) (LOPAP-03, Quma),^33,34^ an NO/NO_2_/NO*_x_* analyzer with a blue light photolytic converter (PLC 860, Eco Physics), and a cavity attenuated phase shift NO_2_ monitor (CAPS–NO_2_, Aerodyne Research, Inc.) for direct NO_2_ detection. Schematic representation of the ACD-C aerosol chamber setup is given in SI.

### Experimental Runs Procedures

Before every experiment, the chamber was flushed overnight with purified air (200 L min^–1^), the air in the chamber was thermostated to 20 °C and, when appropriate, humidified to approximately 80% RH (humid conditions). This was done by flushing the chamber with up to 99% humid air (Nafion-based humidifier was used to produce it) until the required RH was reached. The chamber was closed afterward, and the experiment was started. In the case of experiments performed under dry conditions, the air contained <5% RH.

An organic precursor was introduced into the chamber by slow injection of an aqueous solution of the GUA standard (500 μL in 5 min followed by 5 min flushing) in an inlet with a 200 L min^–1^ stream of purified air. In this way, droplets that contained GUA evaporated immediately and only gaseous GUA entered the chamber (approx. 60 ppb). After GUA injection, the aerosol was generated by 80 s nebulization of a mildly acidic aqueous solution of NaNO_2_ (seed solution; 0.46 g of NaNO_2_ was dissolved in 50 mL of ultrapure Milli-Q water and adjusted to pH = 4.5 with concentrated H_2_SO_4_). Droplets of seed solution were dried immediately after nebulization so that only dry particles of NaNO_2_/H_2_SO_4_ entered the chamber. Every nonblank experiment started with seed introduction, which followed GUA injection and flushing.

A set of experiments under different experimental conditions was conducted: (a) dry dark (DRD; protected from light, <5% RH), (b) humid dark (RHD; protected from light, 80% RH), (c) dry illuminated (DRILL; sunlight bulbs turned on, <5% RH), and (d) humid illuminated (RHILL; sunlight bulbs turned on, 80% RH). Blank experiments were also performed in the absence of either GUA or seed particles to evaluate for direct GUA photolysis and wall losses. Every experiment lasted for 2 h and was followed by air sampling for offline analyses.

### Experimental Methods: Sampling, Sample Preparation, and Analysis

After a chamber experiment had been completed, the lights were turned off (if applicable) and the air was sucked from the chamber (1 h with approx. 30 L min^–1^) through two XAD-4-coated glass denuders (URG, Chapel Hill) with the efficiency to bind volatile organic compounds (VOC) and oxygenated VOC (OVOC) followed by a holder with a poly(tetrafluoroethylene) (PTFE) filter for collecting particles (47 mm, PALL). Only in some additional experiments, Tenax TA cartridges were also used (200 mL min^–1^ for 5 min) for VOC sampling (with a prefilter for particle capture).

Denuders were kept airtight before extraction with 50 mL of methanol, according to the prescribed procedure.^35^ The extraction of the denuder and filter samples was always performed immediately after the sampling. A denuder extract was rotary evaporated (100 mbar at 20 °C) and dried to dryness under a slight stream of nitrogen so that the loss of volatile components was minimized. PM extracts were prepared by extraction with methanol (two times with 1 mL of methanol per filter, 30 min agitation at 500 rpm), filtered through a PTFE syringe filter to remove any particulates, and dried under a slight stream of nitrogen. Only dry samples were stored in a freezer until redissolution, filtration, and analysis.

An Agilent 1100 series high-performance liquid chromatography (HPLC) system coupled with a diode array detector and a Bruker micrOTOF mass spectrometer with electrospray ionization (ESI) were used for the molecular analysis by LC–MS in a negative ionization mode. The separation of phenolic components, including their isomeric forms, was achieved on an Atlantis T3 column (2.1 × 100 mm^2^, 3 μm) with a water/acetonitrile + 0.1% acetic acid V/V gradient starting from 95:5 (kept for 5 min) until 80:20 (5–20 min) and 20:80 (20–30 min), and back down to 95:5 (40–41 min) for 15 min equilibration before the injection of the next sample. The column temperature was set to 25 °C. The flow rate and injection volume were 0.3 mL min^–1^ and 0.5 μL, respectively. Before the analysis, every dry sample was redissolved in 100 μL of methanol and diluted 1:1 with an aqueous solution of 3-nitrobenzoic acid as an internal standard.

Tenax TA cartridges were analyzed by a thermodesorption GC-MS (TurboMatrix 650, PerkinElmer). The gas chromatography–mass spectrometric analysis (GC-MS; 5975C Series GC/MSD, Agilent Technologies) was performed in selected ion monitoring (SIM) mode. A ZB-5ms column (60 m × 0.25 mm × 0.25 μm) was used at a 1.4 mL min^–1^ flow rate. The temperature was increased from 65 to 320 °C by a 10 °C min^–1^ ramp followed by a 7 min temperature increase at 340 °C. A 1 μL pulsed split (25:1) injection at 22.8 psi was applied and 2-trifluoromethylbenzaldehyde was used as an internal standard.

## Results and Discussion

### Reaction System Characterization: Initial Conditions

A multiphase aerosol system in the chamber consisted of: (i) initially gaseous GUA, (ii) dry or wet NaNO_2_ particles, and (iii) a mixture of NO, NO_2_, and HONO gases (NO*_y_*), which all originated from a NaNO_2_/H_2_SO_4_ seed solution. Note at this point that NO*_y_* speciation was an extremely complex task due to multiple interferences influencing especially NO and NO_2_ measurements, which has already been pointed out by Yee et al.^31^ for a similar reaction system. For this reason, different instruments were used to measure NO*_x_* and a series of blank experiments were performed to possibly eliminate biases and correctly define experimental conditions. This, however, is necessary for any atmospheric chamber study where upper-level or even much higher (ppm) concentrations are often used as commonly measured in the field.^36^

Different analyzers all gave different results for NO*_x_* concentrations in the chamber, which also held true for the observed trends and warranted a special caution. However, HONO was selectively measured by the LOPAP technique for which interferences are corrected by a two-channel approach.^33,34^ Potential interferences against nitrite-containing particles were estimated negligible for the small seed particles used (<500 nm), for which the sampling efficiency is ≤1% and can be corrected by the two-channel design of the instrument. Moreover, GUA and HONO do not interfere with the CAPS–NO_2_, which detects NO_2_ by its absorption at 430 nm.^37^ This ensures accurate NO_2_ measurements at least at the beginning of the experiments. On the other hand, the comparison of different instruments suggested that HONO gas and/or particulate nitrite strongly interfere with catalytic converters, substantially influencing either of the signals (NO, NO_2_, or both) depending on the instrument used (data not shown).

Knowing the only source of NO*_y_* species in the chamber, which is acidic aqueous droplets containing NaNO_2_, and HONO aqueous-phase chemistry
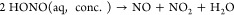
1only the data acquired by the PLC analyzer resulted in the expected equal amounts of NO and NO_2_, characterizing initial reaction conditions as 20–25 ppb NO and NO_2_ in dry air and 15–20 ppb NO and NO_2_ at 80% RH, in addition to 30–40 ppb HONO independent of RH (measured by LOPAP; refer here to Figures S1 and S2). The measured HONO concentrations were an order of magnitude higher than those typically observed in ambient air, therefore the applied conditions can be considered a heavily polluted environment affected by intensive BB events.^28,38^ On the other hand, NO*_x_* concentrations applied are comparable to polluted ambient conditions and much lower than those typically used in high-NO*_x_* chamber studies (i.e., hundreds of ppb to ppm NO*_x_* concentrations).^31^

Despite all of the efforts put in correctly interpreting NO*_x_* data collected with different analyzers, calculations on the nitrogen mass closure still did not fit. As a result, we found out that particulate nitrite, NO, and NO_2_ indeed originated from aqueous droplets containing equilibrium amounts of HONO (NO_2aq_^–^ + H_aq_^+^ ⇌ HONO_aq_) that were sprayed into the chamber, whereas gas-phase HONO must have been additionally added during nebulization. Therefore, we compared the pH of bulk seed solution (H_2_SO_4aq_ ⇌ SO_4__aq_^2–^ + 2 H_aq_^+^) before and after 1 h spraying into the air. It turned out that the pH of the remaining seed solution increased by 1 unit during the course of spraying, which corresponds to the 90% H^+^ loss most likely in the form of HONO (HONO_aq_ ⇌ HONO_g_). Thus, 40 ppb HONO in the chamber is attributed to this process, which also explains why HONO concentration is independent of the experimental conditions, while NO and NO_2_ both decrease at high RH being influenced by the amount of aerosol liquid water.

**Figure 1:**
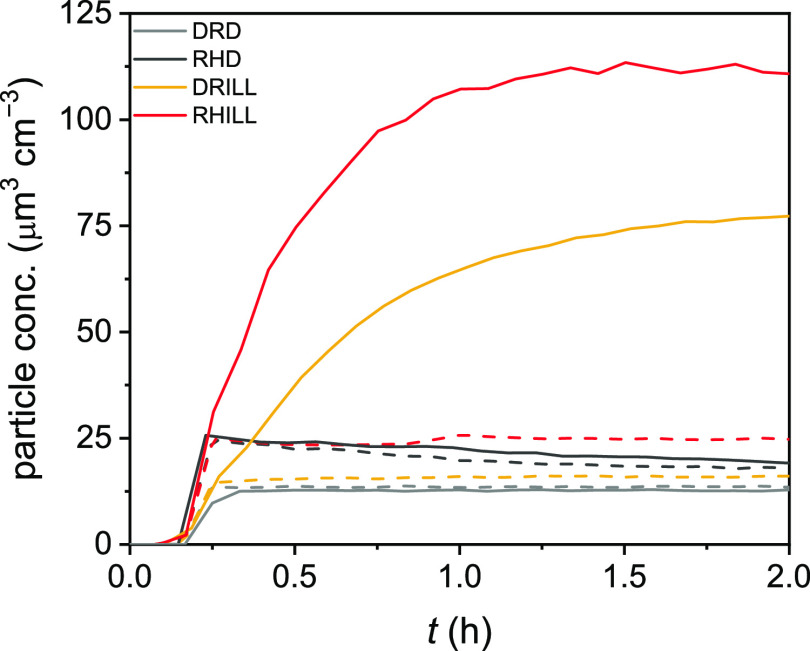
Particulate mass concentration evolution under different conditions: dry dark (DRD), humid dark (RHD), dry illuminated (DRILL), and humid illuminated (RHILL). Dashed lines are blank experiments without guaiacol in the gaseous phase, and solid lines denote experiments with the organic species present.

Although no suitable thermodynamic model exists to estimate the composition (and pH) of generated deliquesced NaNO_2_ particles, the following can be deduced supporting our experimental data. Under dry conditions a much more concentrated aqueous phase is achieved at the beginning of the experiment, resulting in much faster kinetics of reaction 1 and consequently (i) higher concentrations of NO and NO_2_ and (ii) less residual PM mass in the chamber. Consequently, less acidic protons are left in the chamber, which may influence the pH of aerosol liquid water during the experiment (there is a very small amount of liquid water and also less acidic species). On the other hand, at increased RH, droplets are more dilute and HONO_aq_ decomposition according to reaction 1 is slower, leaving more HONO/NO_2_^–^ within the wet aerosol particles and giving (i) lower gas-phase NO and NO_2_ and (ii) larger PM mass. All this is supported by particle measurements presented in Figure 1. At high RH, the measured dry particle mass is always larger than that in dry conditions, which is not due to incomplete drying out before the analysis; see dashed lines for blank experiments.

Furthermore, levels of HONO in simulation chambers have been known to be affected by wall effects. Several different heterogeneous HONO sources have been identified so far, among others a slow heterogeneous dark reaction 2 between NO_2_ and water,^39^ and the conversion of NO_2_ into HONO on light-activated aromatic surface films (reaction 3).^40^
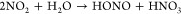
2
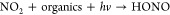
3

The latter process is especially pronounced at increased RH^21,41^ and its effect can be observed as the increase of the HONO concentration during the RHILL experiment, shown in Figure S2d. If this HONO increase is due to reaction 2, which is typically proposed to explain HONO formation in smog chambers, we would also observe the HONO increase during the blank experiment in Figure S1d, which is not the case. Furthermore, the concomitant NO_2_ increase is due to NO to NO_2_ conversion upon organic addition (presumably oxidation by formed peroxy radicals), which overrides NO_2_ consumption via HONO formation.

### Product Analysis and Phase Distribution

**Figure 2:**
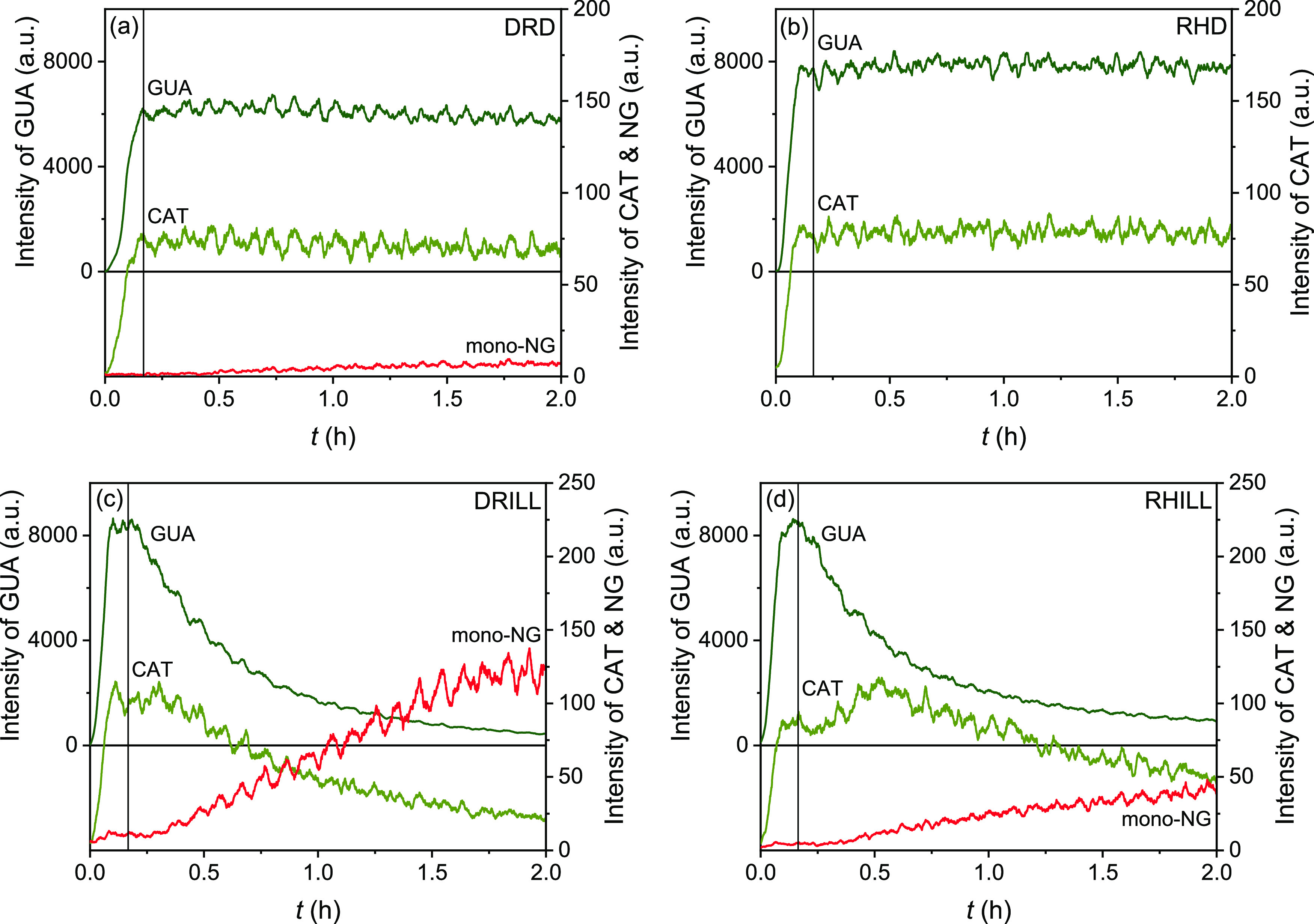
Organic gas mass evolution under different reaction conditions by online PTR-MS: (a) dry dark (DRD), (b) humid dark (RHD), (c) dry illuminated (DRILL), and (d) humid illuminated (RHILL); guaiacol (GUA-H^+^*m*/*z* 125.0603) is a precursor compound and catechol (CAT-H^+^*m*/*z* 111.0446) is its impurity in trace amounts; and isomeric mono-nitroguaiacols (mono-NG-H^+^*m*/*z* 170.0453) are tentatively identified based on their *m*/*z*. Vertical lines denote the start of the experiment by seed injection.

The time series of the gaseous precursor and NG as measured by online PTR-MS is shown in Figure 2. As quantification of nitroaromatic products, in particular, was impossible due to their affinity to stick to the walls (chamber and pipeline), we show relative intensities instead of exact gas concentrations. It is further important to understand that the intensities can only be compared between the same entity and under the same experimental conditions, therefore we cannot deduce any kinetic information from this data.

NG formation was detected under illumination (DRILL and RHILL conditions) and in dry air in the dark (DRD). In the RHD experiment, no NG formation was observed by PTR-MS. In none of the experiments, multiple nitration products were measured, only mono-nitroguaiacols (mono-NG). GUA remained nearly unreacted in the dark, whereas it was almost completely consumed during the course of illuminated experiments. Moreover, the data in Figure 2 show that trace amounts of catechol (CAT) were unintentionally injected in the chamber with the standard solution of GUA. CAT impurities in the order of 1% are estimated from the signal intensities. Additional CAT formation is observed in the RHILL experiment.

Product identification and quantification were further carried out with use of LC–MS and commercially available standards. Extraction efficiency was not evaluated for each specific component; therefore, the results should be considered as lower-limit concentrations. The main nitrated ring-retaining products are gathered in Table S1, together with their gas- and particle-phase concentrations, and the product yields after 2 h of reaction. GUA was confirmed in the denuder samples with the highest concentration in the RHD sample and the lowest concentrations found under illumination (RHD > DRD > DRILL ∼ RHILL; data not shown).

Our product analysis confirmed that GUA photochemistry is closely linked to CAT multiphase chemistry, which has already been observed previously.^30,31^ Besides trace amounts of CAT in the chamber due to impurities in the GUA standard, CAT was additionally formed especially during RHILL experiments (data not shown). Important to note: the chemistry of CAT is thus considered daytime chemistry in this work, which, however, does not necessarily mean that secondary reactions via this pathway require light to be formed.

**Figure 3:**
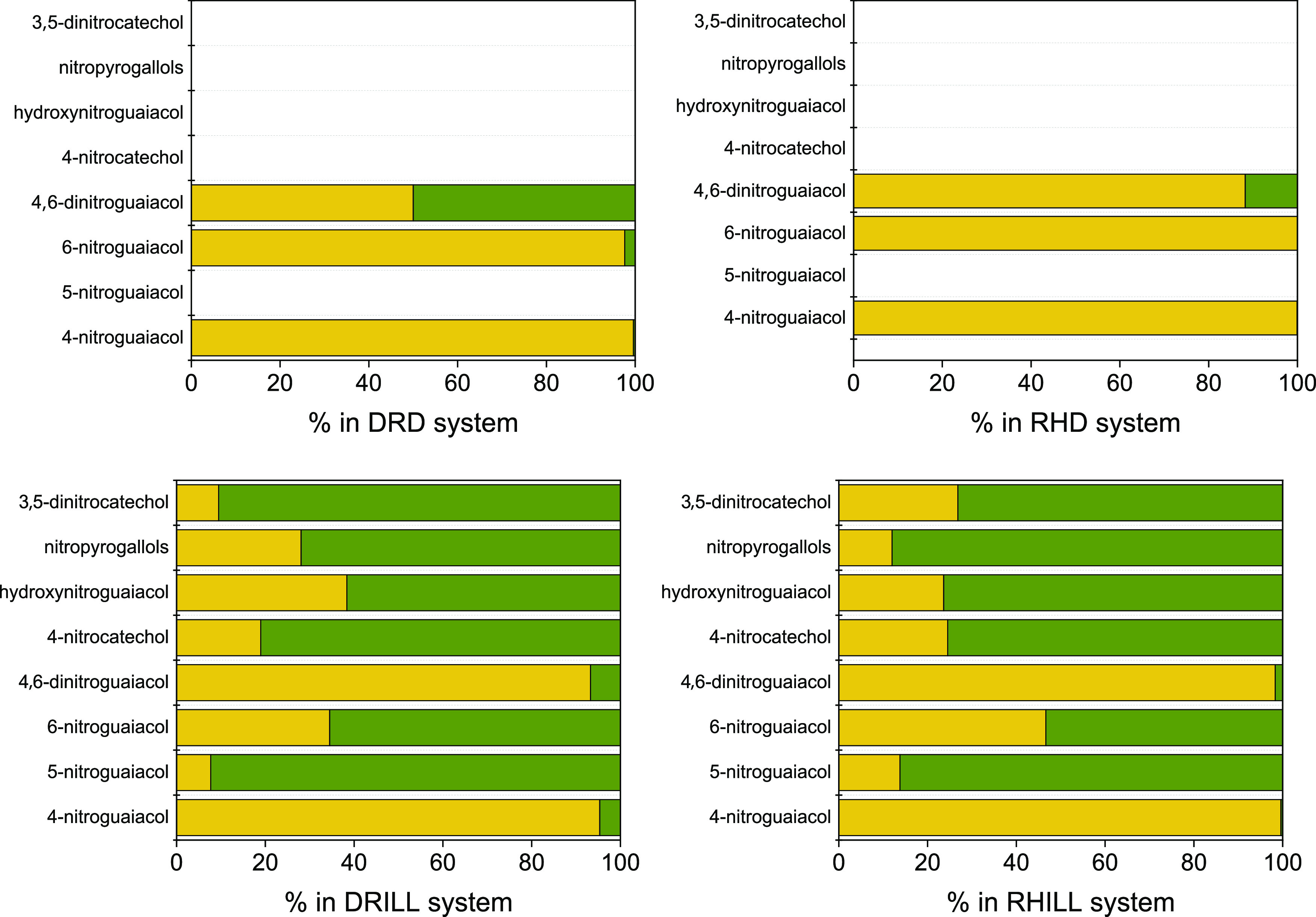
Aromatics distribution in the multiphase system; gas-phase (yellow) and particulate (green) fractions are shown as determined by LC–MS (data taken from Table S1).

The partitioning of major phenols between both phases is presented in Figure 3. In the presence of reactive nitrogen species, NO, NO_2_, HONO, and aqueous HONO/nitrite, initially gaseous GUA is oxidized to various nitration products with the retained aromatic ring, which either remain in the gas or partition to the particulate phase. Although solely GUA nitration increases the product O/C ratio for a factor of 2 (O/C ∼ 0.6), some of those first-generation products still preferentially remain in the gaseous phase (4NG, 4,6DNG). An exception is minor 5NG that was found strongly enriched in the particulate phase. Due to the lack of experimental Henry’s constants (note: theoretical estimations based on group contributions do not distinguish between aromatic isomers), we cannot comment further on the phase distribution of different isomeric nitro compounds.

In comparison to GUA, CAT is much more water-soluble (two orders of magnitude higher Henry’s law constant)^42^ and thus distributed more toward the particulate phase. This increases the possibility of its aqueous-phase aging and implies the potential for aqueous SOA (aqSOA) formation. Ofner et al.^30^ have reported larger SOA yields from CAT than those from GUA, which even increased at high RH. This is consistent with our observations. An important fraction of identified CAT nitration products is found in the particulate phase (e.g., 4NC), contributing to the produced SOA mass. Similar has been observed in ambient air, even on hot summer days.^43^

In general, it can be concluded that although the first-generation products seem to retain some prevalence for the gaseous phase, GUA photooxidation rapidly produces SOA with a high yield (28 and 42% for dry and humid conditions, respectively; the density of 1.45 g cm^–3^ for GUA SOA was used), which is consistent with other studies of this system.^31^ Moreover, we found that sole GUA nitration does not produce substantial SOA mass. Partitioning to the particulate phase is limited even in the case of 4,6DNG with the O/C ratio of 0.85 being comparable with ring-retaining bicyclic peroxides that are usually considered as low-volatile highly oxidized molecules (HOM).^44^ This implies that airborne compounds with high oxygen contents do not necessarily form SOA^45^ but can conversely act as a gas-phase carbon reservoir, which can be ascribed to the decreased reactivity of nitrated aromatics due to deactivating substituent group(s). On the other hand, those products arising from the CAT route (e.g., 4NC) can be important constituents of formed SOA mass and can further substantially contribute to atmospheric absorption by BrC.

### Gas-Phase Reaction Mechanisms

GUA nitration proceeds not only under illumination but also in the dark. As no significant O_3_ formation is observed in any of our dark experiments (Figure S3), this indicates the absence of NO_3_ radical chemistry, which is considered typical for atmospheric nighttime conditions.^17^ On the other hand, OH-assisted nitration was made possible under illumination, in parallel to the major phenolic hydroxylation route^46^ (not discussed here) and the ipso substitution to CAT formation.

The main source of OH in the chamber was the photolysis of HONO, which is also an important source of OH radicals in ambient air.^47,48^ Besides reaction 1 and the partitioning of NO*_y_* from the aqueous phase, the chain of relevant gas-phase reactions 4–7 is experimentally supported by the online measurements. Refer here to Figures S2 and S3, and note that in the absence of organics, O_3_ concentrations of <6 ppb O_3_ are anticipated upon illumination also according to the Leighton relationship.
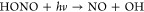
4
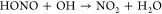
5
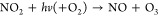
6a
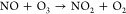
6b
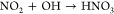
7

**Scheme 1:**
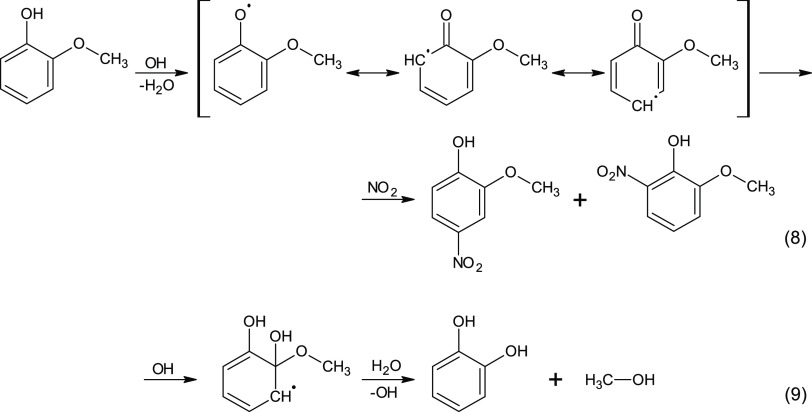
Established Daytime OH-Mediated Radical Chemistry for Gas-Phase Phenols: the Case of Guaiacol Mechanisms of (8) guaiacol nitration and (9) carbon loss are shown.

The established organics daytime chemistry initiated by OH radicals and relevant for our observations is summarized in Scheme 1.

In general, the attack of the OH radical on the phenolic moiety results in either the [Ar–OH]^•^ adduct or substituted Ph^•^ formation. In the atmosphere, the major [Ar–OH]^•^ adduct is believed to preferentially react with O_2_ (not in the focus of this study), whereas minor Ph^•^ can (among others) react with NO_2_ forming isomeric nitration products after an H-atom transfer.^49^ The latter step, however, has not been clarified yet, but likely involves water molecules. Moreover, in the real atmosphere, another source of Ph^•^ is the reaction with nighttime NO_3_ radicals,^10^ which is, however, not relevant for our system (refer here to the discussion above). In the case of Ph^•^ formation, which is a minor pathway of the phenol + OH reaction, two resonance structures are possible, giving 4NG and 6NG products.

To date, there have been several mechanisms proposed for the loss of carbon from substituted benzenes.^31^ The substitution of the methoxy group with a hydroxy group is likely initiated by the *ipso* attack of OH followed by the release of methoxy radicals. It has been suggested very recently by density functional theory (DFT) calculations that the [Ar–OH]^•^ adduct with OH attached at position 2 is most favorable.^50^ Another study showed that although it is theoretically feasible that OH binds to any aromatic C-atom in GUA, giving the corresponding isomeric [Ar–OH]^•^ adducts and products, the OH attack to the hydroxyl-bearing carbon atom and to positions 2 and 4 are slightly more favorable as to the other C-atoms.^24^ Moreover, our study shows that carbon loss is facilitated at high RH, which further implies the involvement of water molecules in the process of methoxy group release, possibly yielding methanol and the OH radical instead of the methoxy radical. Alternatively, carbon loss can also be explained by initial H-abstraction from the methoxy group followed by O_2_ binding and the elimination of formaldehyde in the reductive NO atmosphere.^50^ Neither of those byproducts, however, have been experimentally detected that the exact mechanism could have been unequivocally confirmed.

Once CAT has been formed, it can react analogously to (8) giving the corresponding nitration products to the CAT precursor (CAT pathway). Moreover, the direct formation of 4NC from GUA has also been proposed in a very recent study,^50^ although it is generally believed that [Ar–OH]^•^ adducts do not combine with NO_2_.

**Scheme 2:**
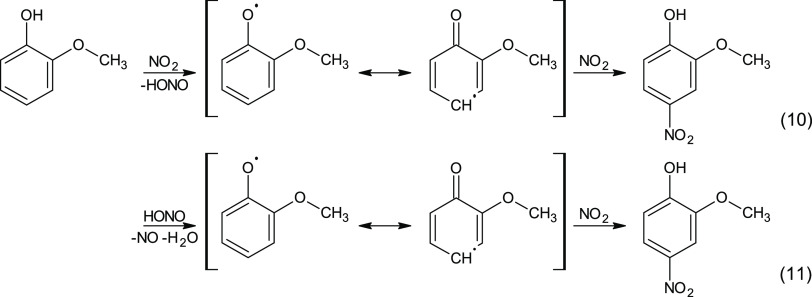
Possible Nighttime Gas-Phase NO*_y_* Chemistry Mechanisms Only the second mechanism (11) supports our observations.

On the other hand, dark aromatic nitration in the absence of NO_3_ radicals has not been established yet. Recently, it has been speculated by Yee et al.^31^ that the direct HONO/NO*_x_* reaction with GUA is possible in the dark nitroaromatic formation. As we only observe it under the dry conditions (Figure 2a), two gas-phase nitration mechanisms are considered for the observed chemistry (Scheme 2).

The first mechanism (10) follows the theoretical calculations performed by Bedini et al.,^51^ which showed that the formation of Ph^•^ solely by NO_2_ is plausible. In aqueous solutions, this type of reaction has been shown to proceed 3–4 orders of magnitude faster with a phenolate ion compared to the protonated molecule.^21,52^ To the best of our knowledge, however, this reaction has never been experimentally confirmed in the gaseous phase, whereas it is possible to proceed heterogeneously on specific substrate surfaces. Woodill et al.^53^ observed 4NC formation by the reaction between NO_2_ and CAT adsorbed on surrogate particles to tropospheric aerosols. Nevertheless, they found a higher 4NC product yield at increased RH (30%), which is contradictory to our observations. Moreover, Guan et al.^54^ suggest a similar reaction mechanism for dark heterogeneous nitration on soot producing nitrated polycyclic aromatic hydrocarbons (PAH) and substantial amounts of HONO. NO_2_ to HONO conversion has also been observed on solid aromatic films, which was shown to be photosensitive and again increased with humidity.^55^ HONO, however, was consumed and not formed during the dark experiment in this study (Figure S1b), preferring the other proposed pathway to be the corresponding mechanism.

The latter proposed mechanism (11), however, again originates from solution chemistry, where HONO has proven itself to be a better oxidant to neutral species than NO_2_.^25,26^ If its oxidative characteristics are retained in the gaseous form, HONO can be an important source of Ph^•^ from aromatic VOC during nighttime. The formed Ph^•^ can then react with NO_2_ in the second step forming the corresponding NP. In this case, the amount of NO formed would be equal to the amount of HONO consumed (i.e., HONO reduction to NO) and the produced equivalent of Ph^•^ would again react with a comparable amount of NO_2_; −ΔNO = ΔHONO = ΔNO_2_ is consistent with our observations (Figure S1b, dashed lines). Furthermore, oxidation by HONO is expected to be more important in the case of better reducing agents such as CAT. In parallel to the nitration of GUA, nitrocatechol (NC) formation from the CAT impurity was indeed observed in the dark experiment (Table S1), giving only a 7-times less 4NC product in comparison to the cumulative concentration of NG in dry air. From the ratio of both precursor compounds (CAT impurity is estimated to be in the order of 1% from the ratio of PTR-MS signals), however, a 100-times lower concentration of 4NC would be anticipated if the reactivity of both compounds, CAT and GUA, toward HONO was the same.

### Heterogeneous Chemistry

Due to a great body of literature on heterogeneous aromatic nitration mentioned above, DRD reaction conditions were additionally investigated for the influence of particles on the observed nitration kinetics. A filter was placed behind the nebulizer where the produced droplets were caught before the aerosol entered the chamber. Consequently, there were no NaNO_2_ particles in the chamber, and according to the CAPS–NO_2_ instrument, only trace amounts of NO_2_ (data not shown; note that by reaction 1 similar amounts of NO are also anticipated). As a result, comparable mono-NG product yields were obtained by thermodesorption GC-MS with and without NaNO_2_ particles (data not shown), which does not favor heterogeneous NO_2_ chemistry, although it cannot be completely excluded due to possible effects of chamber walls. It is important to note at this point that the surface of the chamber walls greatly exceeds the surface area of aerosol particles in the chamber.

Although many of our observations point to the above-proposed homogeneous redox mechanism and suggest that reactions with HONO can be another source of Ph^•^ and NP in the atmosphere, heterogeneous nitration by the nitrosonium ion (NO^+^) on chamber walls followed by oxidation to the corresponding nitro analogue remains a possible mechanism for the observed NG formation. The pH of liquid water in the chamber can be very low especially under dry conditions (only up to 20 g of water is estimated in the chamber), which could trigger the formation of NO^+^ from the protonated HONO upon H_2_O elimination and allow for the electrophilic aromatic substitution reactions to happen. The activation energy of GUA nitrosation in aqueous solution is, however, very high (263 and 309 kJ mol^–1^ for the attack on positions 4 and 6), which results in very small second-order reaction rate constants in the order of 10^2^ L mol^–1^ s^–1^.^23,24^ Nevertheless, besides HONO consumption this mechanism also anticipates NO_2_ to NO conversion, which agrees with our observations and cannot be completely excluded. This, however, warrants further investigation.

### Aqueous-Phase Aging

**Scheme 3:**
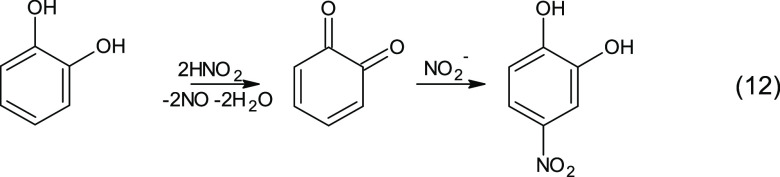
Nighttime Aqueous-Phase Nitration of Catechol as Proposed by Vidović et al.^25,26^

At increased RH, 50% higher SOA yields were observed than under the dry conditions (compare DRILL and RHILL in Figure 1), which implies aqSOA formation from aromatic precursors when humid conditions are applied. Among the identified products with retained aromaticity, especially nitrated CAT analogues (4NC and nitrated pyrogallol; Table S1) were enriched in the particulate phase at high RH. This can be explained by the very recently proposed aqueous-phase CAT chemistry to NC formation at moderately acidic pH, which proceeds by HONO oxidation to the corresponding *o*-quinone and the consequent conjugated addition reaction with nitrite (12). Although this is a dark reaction mechanism, it is still active in irradiated conditions and as CAT forms in daytime chemistry in our case, we could only observe those products in illuminated samples. The reaction is schematically presented in Scheme 3; for the exact mechanism, see Vidović et al.^25,26^

Intriguing is also particulate 6NG in illuminated samples (especially large amounts determined in RHILL samples), which can be attributed to its sunlight-assisted aqueous-phase formation or reactive uptake into the aqueous phase during illumination (note: 6NG is a gas-phase product in the dark; see Figure 3). Although there has been an extensive investigation performed on aqueous-phase GUA nitration,^23,24,56^ those results do not unequivocally support observations in this study. Comparable amounts of 4NG and 6NG are anticipated according to the bulk-solution studies, whereas we observe solely the enrichment of 6NG and only trace amounts of 4NG in the particulate phase.

### Absorption Characteristics of the Extracts

In Figure S4, we show the absorption spectra of the different methanolic extracts gathered in this study. Usually, only SOA mass is extracted and discussed in terms of BrC characteristics, whereas we want to compare denuder and filter extracts and use those data in support of the proposed nitration mechanisms.

In general, the most absorbing were RHILL extracts, followed by DRILL. Under illumination, more absorbing products were captured in the particulate phase (particles vs gas ∼ 2). In the dark, BrC absorption was only measured in the case of the DRD extract, whereas the RHD filter extract remained (also visually) transparent. Moreover, also more absorbing gas-phase products formed under the dry conditions in the dark, exhibiting significant absorption all the way to 500 nm. This observation supports the above-proposed mechanism of dark gas-phase GUA oxidation by HONO to Ph^•^. In the proposed heterogeneous chemistry on chamber walls, only different NG isomers can form via the electrophilic aromatic nitrosation–oxidation mechanism. Those, however, all absorb light below 450 nm (Figure S5). For the extended absorption in the visible region (>450 nm), expansion of the conjugated system is required, which could well result from the recombination of nitrated Ph^•^ species by the HONO oxidation mechanism. Slikboer et al.^57^ observed strong absorption between 400 and 500 nm attributed to the polymerization of GUA. Similar products have been recently observed in the dark GUA nitration experiments by NO_3_ and confirmed in real BB-affected PM samples.^16^

### Environmental Relevance

In this study, HONO-assisted phenolic nitration was investigated in a multiphase system of the ACD-C aerosol chamber, to gain more understanding of the mechanisms of the secondary nitrophenol, and aqSOA and BrC formation from BB precursors in the atmosphere. In an aerosol chamber, as well as in the atmosphere, semivolatile GUA is mostly a gaseous precursor compound, and its first-generation nitrated products partition between both phases dependent on the isomeric form, which results in their limited contribution to BrC.

In contrast to its dark-experiment partitioning, we observed 6NG enriched in the particulate phase under illumination, whereas its formation was pronounced at high RH. This could be attributed to the sunlight-assisted aqueous-phase GUA processing, as proposed recently.^23^ Nevertheless, it is hard to judge whether particulate 6NG in fact resulted from the aqueous-phase reaction, which is also known to yield 4NG, as gas-to-particle partitioning constants are not known for the different isomeric NG.

On the other hand, we observe that GUA hydroxylation or a carbon loss tend to move the multiphase equilibrium toward the particulate phase, which implies an improved potential to form aqSOA and BrC in the atmosphere. 4NC was the prevailing ring-retaining product found in the particulate phase with a product yield of 3.6%, followed by nitrated pyrogallol analogues. All of these are strongly absorbing species in the near-UV and visible ranges and could importantly contribute to BrC absorption below 450 nm. The loss of the methoxy substituent from the ring is evidently linked with high RH and irradiation, however, secondary reactions to NC products could also proceed via the dark, possibly aqueous-phase mechanisms, such as proposed recently.^25^ Due to the possible important adverse effects to the climate and human health, unequivocal confirmation of aqSOA/BrC formation directly from CAT in the presence of HONO warrants further investigation.

In contrast to the general belief, we show that dark NG formation is also possible in the absence of NO_3_, which is repressed at high RH. Based on our observations, we propose a new dark gas-phase mechanism involving initial GUA oxidation by HONO to Ph^•^ and subsequent nitration by NO_2_, although chamber wall chemistry could not be unconditionally excluded. The estimated second-order rate constant for the observed dark NG formation in dry air is in the order of 10^–18^ cm^3^ molecule^–1^ s^–1^, which is roughly six orders of magnitude slower than its competitive OH and NO_3_ radical reactions with GUA.^58^ Although typical atmospheric concentrations of OH and NO_3_ radicals are in the ppt range,^59^ whereas ppb level HONO concentrations can be reached in extremely polluted environments,^60^ the observed dark nitration mechanism is still expected to be a minor pathway of GUA transformation in the environment.

**Scheme 4:**
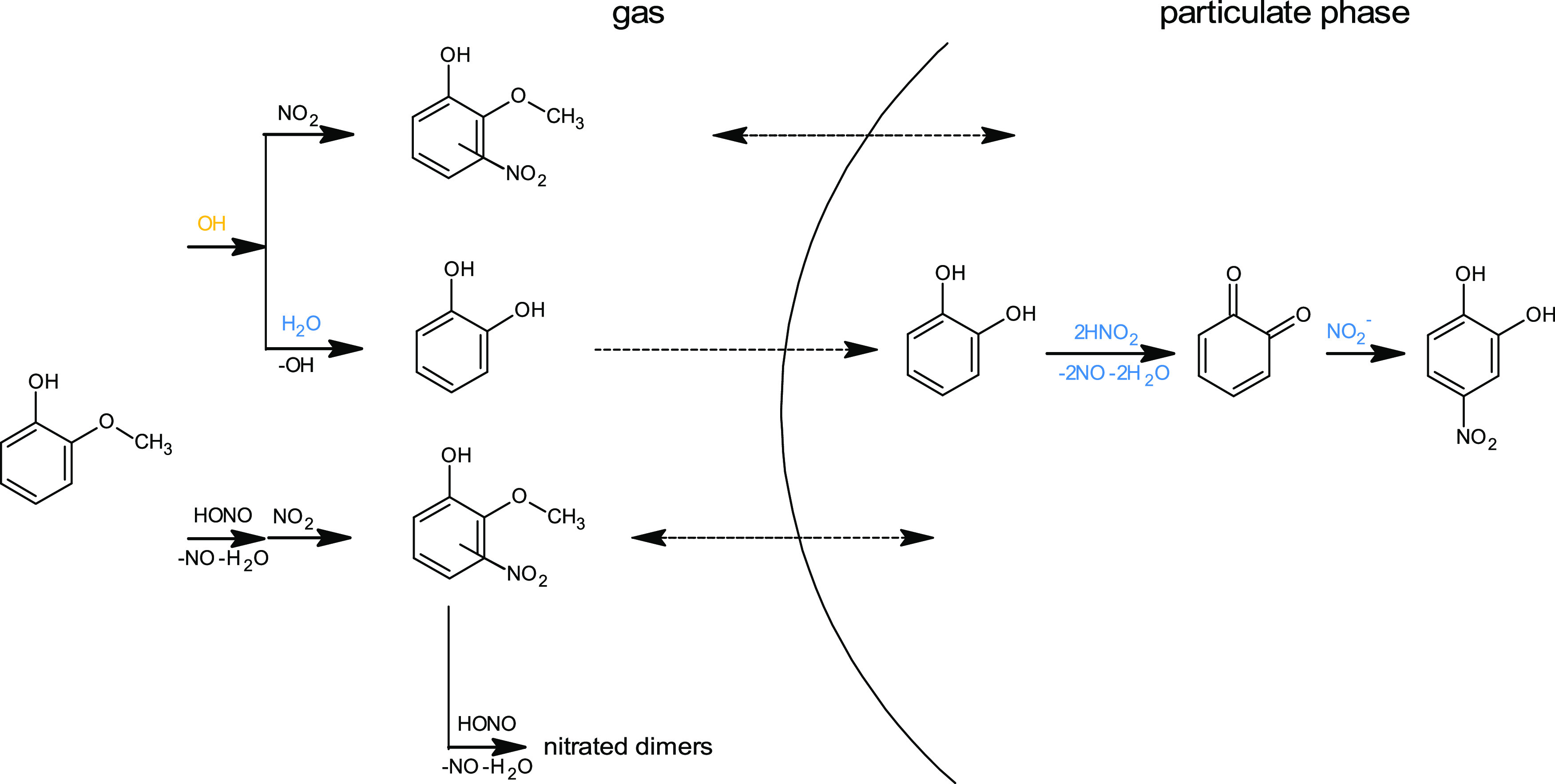
Schematic of the Main Pathways of Multiphase Guaiacol Nitration in the Presence of HONO Only the products that retained aromaticity are considered.

The results presented herein apply to emissions from incomplete combustion of lignocellulosic biomass that are rich in GUA and other methoxyphenol species and HONO; e.g., smoke produced during wood smoldering by natural fire or anthropogenic BB. The main nitration pathways as observed in this study are presented in Scheme 4.

## Supporting Information Available

Experimental setup; synthesis procedure; NO*_y_* and O_3_ time profiles; product quantification and product yields; and absorption spectra (PDF)

## References

[ref1] Abatzoglou J. T.; Williams A. P. Impact of anthropogenic climate change on wildfire across western US forests. Proc. Natl. Acad. Sci. U.S.A. 2016, 113, 11770–11775. 10.1073/pnas.1607171113. 27791053 PMC5081637

[ref2] Mok J.; Krotkov N. A.; Arola A.; Torres O.; Jethva H.; Andrade M.; Labow G.; Eck T. F.; Li Z. Q.; Dickerson R. R.; Stenchikov G. L.; Osipov S.; Ren X. R. Impacts of brown carbon from biomass burning on surface UV and ozone photochemistry in the Amazon Basin. Sci. Rep. 2016, 6, 36940 10.1038/srep36940. 27833145 PMC5105132

[ref3] Brown H.; Liu X. H.; Feng Y.; Jiang Y. Q.; Wu M. X.; Lu Z.; Wu C. L.; Murphy S.; Pokhrel R. Radiative effect and climate impacts of brown carbon with the Community Atmosphere Model (CAM5). Atmos. Chem. Phys. 2018, 18, 17745–17768. 10.5194/acp-18-17745-2018.

[ref4] Wong J. P. S.; Tsagkaraki M.; Tsiodra I.; Mihalopoulos N.; Violaki K.; Kanakidou M.; Sciare J.; Nenes A.; Weber R. J. Atmospheric evolution of molecular-weight-separated brown carbon from biomass burning. Atmos. Chem. Phys. 2019, 19, 7319–7334. 10.5194/acp-19-7319-2019.

[ref5] Mohr C.; Lopez-Hilfiker F. D.; Zotter P.; Prévôt A. S. H.; Xu L.; Ng N. L.; Herndon S. C.; Williams L. R.; Franklin J. P.; Zahniser M. S.; Worsnop D. R.; Knighton W. B.; Aiken A. C.; Gorkowski K. J.; Dubey M. K.; Allan J. D.; Thornton J. A. Contribution of nitrated phenols to wood burning brown carbon light absorption in Detling, United Kingdom during winter time. Environ. Sci. Technol. 2013, 47, 6316–6324. 10.1021/es400683v. 23710733

[ref6] Zhang X. L.; Lin Y. H.; Surratt J. D.; Weber R. J. Sources, composition and absorption Ångström exponent of light-absorbing organic components in aerosol extracts from the Los Angeles basin. Environ. Sci. Technol. 2013, 47, 3685–3693. 10.1021/es305047b. 23506531

[ref7] Laskin A.; Laskin J.; Nizkorodov S. A. Chemistry of atmospheric brown carbon. Chem. Rev. 2015, 115, 4335–4382. 10.1021/cr5006167. 25716026

[ref8] Teich M.; van Pinxteren D.; Wang M.; Kecorius S.; Wang Z. B.; Müller T.; Močnik G.; Herrmann H. Contributions of nitrated aromatic compounds to the light absorption of water-soluble and particulate brown carbon in different atmospheric environments in Germany and China. Atmos. Chem. Phys. 2017, 17, 1653–1672. 10.5194/acp-17-1653-2017.

[ref9] Lin P.; Bluvshtein N.; Rudich Y.; Nizkorodov S. A.; Laskin J.; Laskin A. Molecular chemistry of atmospheric brown carbon inferred from a nationwide biomass burning event. Environ. Sci. Technol. 2017, 51, 11561–11570. 10.1021/acs.est.7b02276. 28759227

[ref10] Yuan B.; Liggio J.; Wentzell J.; Li S. M.; Stark H.; Roberts J. M.; Gilman J.; Lerner B.; Warneke C.; Li R.; Leithead A.; Osthoff H. D.; Wild R.; Brown S. S.; de Gouw J. A. Secondary formation of nitrated phenols: insights from observations during the Uintah BasinWinter Ozone Study (UBWOS) 2014. Atmos. Chem. Phys. 2016, 16, 2139–2153. 10.5194/acp-16-2139-2016.

[ref11] Finewax Z.; de Gouw J. A.; Ziemann P. J. Identification and quantification of 4-nitrocatechol formed from OH and NO3 radical-initiated reactions of catechol in air in the presence of NOx: Implications for secondary organic aerosol formation from biomass burning. Environ. Sci. Technol. 2018, 52, 1981–1989. 10.1021/acs.est.7b05864. 29353485

[ref12] Wang Y.; Hu M.; Wang Y.; Zheng J.; Shang D.; Yang Y.; Liu Y.; Li X.; Tang R.; Zhu W.; Du Z.; Wu Y.; Guo S.; Wu Z.; Lou S.; Hallquist M.; Yu J. Z. The formation of nitro-aromatic compounds under high NOx and anthropogenic VOC conditions in urban Beijing, China. Atmos. Chem. Phys. 2019, 19, 7649–7665. 10.5194/acp-19-7649-2019.

[ref13] Zhao R.; Lee A. K. Y.; Huang L.; Li X.; Yang F.; Abbatt J. P. D. Photochemical processing of aqueous atmospheric brown carbon. Atmos. Chem. Phys. 2015, 15, 6087–6100. 10.5194/acp-15-6087-2015.

[ref14] Barsotti F.; Bartels-Rausch T.; De Laurentiis E.; Ammann M.; Brigante M.; Mailhot G.; Maurino V.; Minero C.; Vione D. Photochemical Formation of Nitrite and Nitrous Acid (HONO) upon Irradiation of Nitrophenols in Aqueous Solution and in Viscous Secondary Organic Aerosol Proxy. Environ. Sci. Technol. 2017, 51, 7486–7495. 10.1021/acs.est.7b01397. 28581723

[ref15] Hems R. F.; Abbatt J. P. D. Aqueous phase photo-oxidation of brown carbon nitrophenols: reaction kinetics, mechanism, and evolution of light absorption. ACS Earth Space Chem. 2018, 2, 225–234. 10.1021/acsearthspacechem.7b00123.

[ref16] Mayorga R. J.; Zhao Z. X.; Zhang H. F. Formation of secondary organic aerosol from nitrate radical oxidation of phenolic VOCs: Implications for nitration mechanisms and brown carbon formation. Atmos. Environ. 2021, 244, 117910 10.1016/j.atmosenv.2020.117910.

[ref17] Liu C. G.; Zhang P.; Wang Y. F.; Yang B.; Shu J. N. Heterogeneous reactions of particulate methoxyphenols with NO3 radicals: kinetics, products, and mechanisms. Environ. Sci. Technol. 2012, 46, 13262–13269. 10.1021/es303889z. 23171305

[ref18] Li C. L.; He Q. F.; Hettiyadura A. P. S.; Käfer U.; Shmul G.; Meidan D.; Zimmermann R.; Brown S. S.; George C.; Laskin A.; Rudich Y. Formation of secondary brown carbon in biomass burning aerosol proxies through NO3 radical reactions. Environ. Sci. Technol. 2020, 54, 1395–1405. 10.1021/acs.est.9b05641. 31730747

[ref19] Barletta B.; Bolzacchini E.; Meinardi S.; Orlandi M.; Rindone B. The NO3 radical-mediated liquid phase nitration of phenols with nitrogen dioxide. Environ. Sci. Technol. 2000, 34, 2224–2230. 10.1021/es990844m.

[ref20] Barzaghi P.; Herrmann H. Kinetics and mechanisms of reactions of the nitrate radical (NO3) with substituted phenols in aqueous solution. Phys. Chem. Chem. Phys. 2004, 6, 5379–5388. 10.1039/b412933d.

[ref21] Ammann M.; Rössler E.; Strekowski R.; George C. Nitrogen dioxide multiphase chemistry: Uptake kinetics on aqueous solutions containing phenolic compounds. Phys. Chem. Chem. Phys. 2005, 7, 2513–2518. 10.1039/b501808k. 15962037

[ref22] Minero C.; Bono F.; Rubertelli F.; Pavino D.; Maurino V.; Pelizzetti E.; Vione D. On the effect of pH in aromatic photonitration upon nitrate photolysis. Chemosphere 2007, 66, 650–656. 10.1016/j.chemosphere.2006.07.082. 16996108

[ref23] Kroflič A.; Grilc M.; Grgić I. Unraveling pathways of guaiacol nitration in atmospheric waters: Nitrite, a source of reactive nitronium ion in the atmosphere. Environ. Sci. Technol. 2015, 49, 9150–9158. 10.1021/acs.est.5b01811. 26162010

[ref24] Kroflič A.; Huš M.; Grilc M.; Grgić I. Underappreciated and complex role of nitrous acid in aromatic nitration under mild environmental conditions: The case of activated methoxyphenols. Environ. Sci. Technol. 2018, 52, 13756–13765. 10.1021/acs.est.8b01903. 30388370

[ref25] Vidović K.; Jurković D. L.; Šala M.; Kroflič A.; Grgić I. Nighttime aqueous phase-formation of nitrocatechols (1,2 dihydroxynitrobenzenes) in the atmospheric condensed phase. Environ. Sci. Technol. 2018, 52, 9722–9730. 10.1021/acs.est.8b01161. 29944831

[ref26] Vidović K.; Kroflič A.; Jovanovič P.; Šala M.; Grgić I. Electrochemistry as a tool for studies of complex reaction mechanisms: The case of the atmospheric aqueous-phase aging of catechols. Environ. Sci. Technol. 2019, 53, 11195–11203. 10.1021/acs.est.9b02456. 31482713

[ref27] Kitanovski Z.; Grgić I.; Vermeylen R.; Claeys M.; Maenhaut W. Liquid chromatography tandem mass spectrometry method for characterization of monoaromatic nitro-compounds in atmospheric particulate matter. J. Chromatogr. A 2012, 1268, 35–43. 10.1016/j.chroma.2012.10.021. 23122275

[ref28] Bluvshtein N.; Lin P.; Flores J. M.; Segev L.; Mazar Y.; Tas E.; Snider G.; Weagle C.; Brown S. S.; Laskin A.; Rudich Y. Broadband optical properties of biomass-burning aerosol and identification of brown carbon chromophores. J. Geophys. Res.: Atmos. 2017, 122, 5441–5456. 10.1002/2016JD026230.

[ref29] Sun Y. L.; Zhang Q.; Anastasio C.; Sun J. Insights into secondary organic aerosol formed via aqueous-phase reactions of phenolic compounds based on high resolution mass spectrometry. Atmos. Chem. Phys. 2010, 10, 4809–4822. 10.5194/acp-10-4809-2010.

[ref30] Ofner J.; Krüger H. U.; Grothe H.; Schmitt-Kopplin P.; Whitmore K.; Zetzsch C. Physico-chemical characterization of SOA derived from catechol and guaiacol – a model substance for the aromatic fraction of atmospheric HULIS. Atmos. Chem. Phys. 2011, 11, 1–15. 10.5194/acp-11-1-2011.

[ref31] Yee L. D.; Kautzman K. E.; Loza C. L.; Schilling K. A.; Coggon M. M.; Chhabra P. S.; Chan M. N.; Chan A. W. H.; Hersey S. P.; Crounse J. D.; Wennberg P. O.; Flagan R. C.; Seinfeld J. H. Secondary organic aerosol formation from biomass burning intermediates: phenol and methoxyphenols. Atmos. Chem. Phys. 2013, 13, 8019–8043. 10.5194/acp-13-8019-2013.

[ref32] Yu L.; Smith J.; Laskin A.; Anastasio C.; Laskin J.; Zhang Q. Chemical characterization of SOA formed from aqueous-phase reactions of phenols with the triplet excited state of carbonyl and hydroxyl radical. Atmos. Chem. Phys. 2014, 14, 13801–13816. 10.5194/acp-14-13801-2014.

[ref33] Heland J.; Kleffmann J.; Kurtenbach R.; Wiesen P. A new instrument to measure gaseous nitrous acid (HONO) in the atmosphere. Environ. Sci. Technol. 2001, 35, 3207–3212. 10.1021/es000303t. 11506004

[ref34] Kleffmann J.; Lörzer J. C.; Wiesen P.; Kern C.; Trick S.; Volkamer R.; Rodenas M.; Wirtz K. Intercomparison of the DOAS and LOPAP techniques for the detection of nitrous acid (HONO). Atmos. Environ. 2006, 40, 3640–3652. 10.1016/j.atmosenv.2006.03.027.

[ref35] Kahnt A.; Iinuma Y.; Böge O.; Mutzel A.; Herrmann H. Denuder sampling techniques for the determination of gas-phase carbonyl compounds: A comparison and characterisation of in situ and ex situ derivatisation methods. J. Chromatogr. B: Anal. Technol. Biomed. Life Sci. 2011, 879, 1402–1411. 10.1016/j.jchromb.2011.02.028. 21411383

[ref36] Villena G.; Bejan I.; Kurtenbach R.; Wiesen P.; Kleffmann J. Interferences of commercial NO2 instruments in the urban atmosphere and in a smog chamber. Atmos. Meas. Tech. 2012, 5, 149–159. 10.5194/amt-5-149-2012.

[ref37] Kebabian P. L.; Herndon S. C.; Freedman A. Detection of nitrogen dioxide by cavity attenuated phase shift spectroscopy. Anal. Chem. 2005, 77, 724–728. 10.1021/ac048715y. 15649079

[ref38] Iinuma Y.; Böge O.; Gräfe R.; Herrmann H. Methyl-nitrocatechols: Atmospheric tracer compounds for biomass burning secondary organic aerosols. Environ. Sci. Technol. 2010, 44, 8453–8459. 10.1021/es102938a. 20964362

[ref39] Finlayson-Pitts B. J.; Wingen L. M.; Sumner A. L.; Syomin D.; Ramazan K. A. The heterogeneous hydrolysis of NO2 in laboratory systems and in outdoor and indoor atmospheres: An integrated mechanism. Phys. Chem. Chem. Phys. 2003, 5, 223–242. 10.1039/b208564j.

[ref40] Stemmler K.; Ammann M.; Donders C.; Kleffmann J.; George C. Photosensitized reduction of nitrogen dioxide on humic acid as a source of nitrous acid. Nature 2006, 440, 195–198. 10.1038/nature04603. 16525469

[ref41] Han C.; Yang W. J.; Wu Q. Q.; Yang H.; Xue X. X. Heterogeneous photochemical conversion of NO2 to HONO on the humic acid surface under simulated sunlight. Environ. Sci. Technol. 2016, 50, 5017–5023. 10.1021/acs.est.5b05101. 27074517

[ref42] Sander R. Compilation of Henry’s law constants (version 4.0) for water as solvent. Atmos. Chem. Phys. 2015, 15, 4399–4981. 10.5194/acp-15-4399-2015.

[ref43] Li M.; Wang X. F.; Lu C. Y.; Li R.; Zhang J.; Dong S. W.; Yang L. X.; Xue L. K.; Chen J. M.; Wang W. X. Nitrated phenols and the phenolic precursors in the atmosphere in urban Jinan, China. Sci. Total Environ. 2020, 714, 136760 10.1016/j.scitotenv.2020.136760. 31982756

[ref44] Wang S. N.; Wu R. R.; Berndt T.; Ehn M.; Wang L. M. Formation of highly oxidized radicals and multifunctional products from the atmospheric oxidation of alkylbenzenes. Environ. Sci. Technol. 2017, 51, 8442–8449. 10.1021/acs.est.7b02374. 28682596

[ref45] Bianchi F.; Kurtén T.; Riva M.; Mohr C.; Rissanen M. P.; Roldin P.; Berndt T.; Crounse J. D.; Wennberg P. O.; Mentel T. F.; Wildt J.; Junninen H.; Jokinen T.; Kulmala M.; Worsnop D. R.; Thornton J. A.; Donahue N.; Kjaergaard H. G.; Ehn M. Highly oxygenated organic molecules (HOM) from gas-phase autoxidation involving peroxy radicals: A key contributor to atmospheric aerosol. Chem. Rev. 2019, 119, 3472–3509. 10.1021/acs.chemrev.8b00395. 30799608 PMC6439441

[ref46] Olariu R. I.; Klotz B.; Barnes I.; Becker K. H.; Mocanu R. FT-IR study of the ring-retaining products from the reaction of OH radicals with phenol, o-, m-, and p-cresol. Atmos. Environ. 2002, 36, 3685–3697. 10.1016/S1352-2310(02)00202-9.

[ref47] Alicke B.; Platt U.; Stutz J. Impact of nitrous acid photolysis on the total hydroxyl radical budget during the Limitation of Oxidant Production/Pianura Padana Produzione di Ozono study in Milan. J. Geophys. Res.: Atmos. 2002, 107, 8196 10.1029/2000JD000075.

[ref48] Kleffmann J.; Gavriloaiei T.; Hofzumahaus A.; Holland F.; Koppmann R.; Rupp L.; Schlosser E.; Siese M.; Wahner A. Daytime formation of nitrous acid: A major source of OH radicals in a forest. Geophys. Res. Lett. 2005, 32, L05818 10.1029/2005GL022524.

[ref49] Berndt T.; Böge O. Gas-phase reaction of OH radicals with phenol. Phys. Chem. Chem. Phys. 2003, 5, 342–350. 10.1039/B208187C.

[ref50] Sun Y. H.; Xu F.; Li X. F.; Zhang Q. Z.; Gu Y. X. Mechanisms and kinetic studies of OH-initiated atmospheric oxidation of methoxyphenols in the presence of O2 and NOx. Phys. Chem. Chem. Phys. 2019, 21, 21856–21866. 10.1039/C9CP03246K. 31553018

[ref51] Bedini A.; Maurino V.; Minero C.; Vione D. Theoretical and experimental evidence of the photonitration pathway of phenol and 4-chlorophenol: A mechanistic study of environmental significance. Photochem. Photobiol. Sci. 2012, 11, 418–424. 10.1039/C1PP05288H. 22124765

[ref52] Umschlag T.; Zellner R.; Herrmann H. Laser-based studies of NO3 radical reactions with selected aromatic compounds in aqueous solution. Phys. Chem. Chem. Phys. 2002, 4, 2975–2982. 10.1039/b110263j.

[ref53] Woodill L. A.; Hinrichs R. Z. Heterogeneous reactions of surface-adsorbed catechol with nitrogen dioxide: substrate effects for tropospheric aerosol surrogates. Phys. Chem. Chem. Phys. 2010, 12, 10766–10774. 10.1039/c002079f. 20623042

[ref54] Guan C.; Li X. L.; Zhang W. G.; Huang Z. Identification of nitration products during heterogeneous reaction of NO_2_ on soot in the dark and under simulated sunlight. J. Phys. Chem. A 2017, 121, 482–492. 10.1021/acs.jpca.6b08982. 28005389

[ref55] George C.; Strekowski R. S.; Kleffmann J.; Stemmler K.; Ammann M. Photoenhanced uptake of gaseous NO_2_ on solid organic compounds: a photochemical source of HONO?. Faraday Discuss. 2005, 130, 195–210. 10.1039/b417888m. 16161785

[ref56] Kroflič A.; Grilc M.; Grgić I. Does toxicity of aromatic pollutants increase under remote atmospheric conditions?. Sci. Rep. 2015, 5, 8859 10.1038/srep08859. 25748923 PMC4352892

[ref57] Slikboer S.; Grandy L.; Blair S. L.; Nizkorodov S. A.; Smith R. W.; Al-Abadleh H. A. Formation of light absorbing soluble secondary organics and insoluble polymeric particles from the dark reaction of catechol and guaiacol with Fe(III). Environ. Sci. Technol. 2015, 49, 7793–7801. 10.1021/acs.est.5b01032. 26039867

[ref58] NIST Chemical Kinetics Database. In Vol. Standard Reference Database 17, Version 7.0 (Web Version), Release 1.6.8, Data Version 2015.09.

[ref59] Seinfeld J. H.; Pandis S. N. Atmospheric Chemistry and Physics: From Air Pollution to Climate Change.; John Wiley & Sons, 2016.

[ref60] Zhang L.; Wang T.; Zhang Q.; Zheng J. Y.; Xu Z.; Lv M. Y. Potential sources of nitrous acid (HONO) and their impacts on ozone: A WRF-Chem study in a polluted subtropical region. J. Geophys. Res.: Atmos. 2016, 121, 3645–3662. 10.1002/2015JD024468.

