# Supplementary material for: Guaiacol Nitration in a Simulated Atmospheric Aerosol with an Emphasis on Atmospheric Nitrophenol Formation Mechanisms
Source: ACS Earth Space Chem. 2021 Apr 12;5(5):1083–93. doi: 10.1021/acsearthspacechem.1c00014 (PMC8161671; doi:10.1021/acsearthspacechem.1c00014)
Supplement: Supplementary file 1 — sp1c00014_si_001.pdf [file sp1c00014_si_001.pdf]

Supporting Information to

Guaiacol Nitration in a Simulated Atmospheric Aerosol with an Emphasis on Atmospheric Nitrophenol Formation Mechanisms

Ana Kroflič,[a,b,*] Janine Anders,[b] Ivana Drventić,[a] Peter Mettke,[b] Olaf Böge,[b] Anke Mutzel,[b] Jörg Kleffmann[c] and Hartmut Herrmann[b,*]

[a]Department of Analytical Chemistry, National Institute of Chemistry, Hajdrihova 19, 1000 Ljubljana, Slovenia

[b]Leibniz-Institute for Tropospheric Research (TROPOS), Atmospheric Chemistry Department (ACD), Permoserstrasse 15, 04318 Leipzig, Germany

[c]Physical and Theoretical Chemistry, University of Wuppertal, Gaußstrasse 20, 42119 Wuppertal, Germany

Corresponding Authors

E-mail: ana.kroflic@ki.si (A.K.), herrmann@tropos.de (H.H.)

Supporting information includes:

9 pages, 1 Scheme, 5 Figures, 1 Table

ACD-C ATMOSPHERIC CHAMBER SETUP

The experiments were performed in the ACD-C aerosol chamber at TROPOS, Leipzig. A clean air generator was used to purify ambient air for the purpose of atmospheric chamber operation and cleaning. Additionally, a nafion-based air humidifier was used for high-RH experiments. After the desired experimental conditions (T and RH) had been reached, the chamber was kept closed during the experiment.

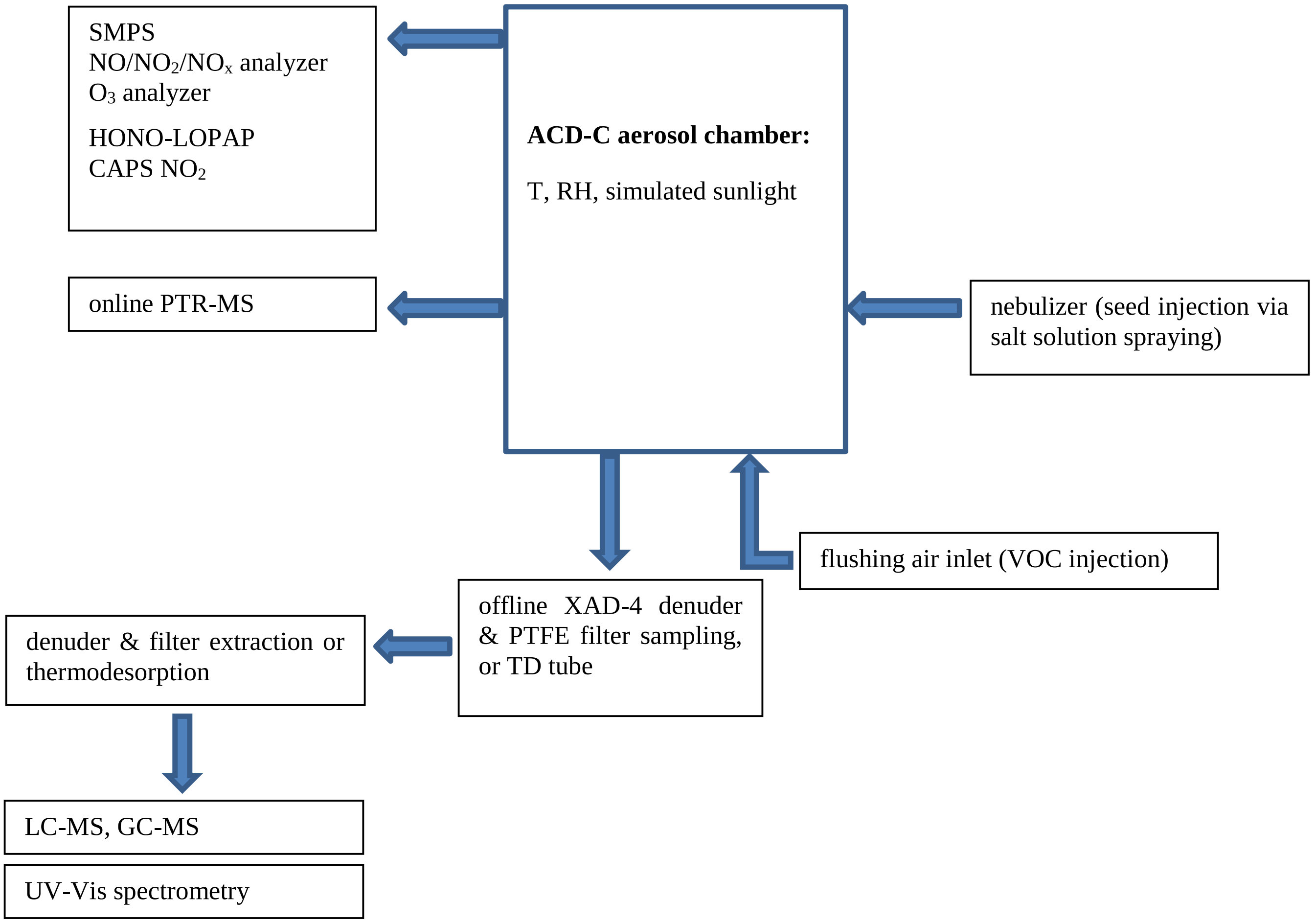

Scheme S1. Experimental setup of ACD-C aerosol simulation chamber as used in this study.

DE NOVO SYNTHESIS PROCEDURE

In a 5 L round-bottom flask equipped with a mercury-vapor lamp, 44.1 mg (0.35 mmol; 1 eqv.) pyrogallol (GAL) was dissolved in 3.5 L purified water. Subsequently, 241.5 mg (3.5 mmol; 10 eqv.) of sodium nitrite and 396.9 mg of a hydrogen peroxide solution (30%; 3.5 mmol; 10 eqv.) were added. The reaction mixture was illuminated for 4 h. Samples were taken at the beginning of the experiment as well as after 2 and 4 h of illumination. The sample aliquots were analyzed by LC-MS without further purification. A compound with the same retention time and the same mass-to-charge ratio was also observed in the chamber experiments.

Further synthetic efforts were performed to obtain pure analytical standard. Nitration by ammonium nitrate with both acetic anhydride and trifluoroacetic anhydride[1] in dichlormethane did not result in the formation of nitropyrogallol, but rather led to a rapid decomposition of the polyphenolic compound. Moreover, the isolation of the nitrated GAL from the reaction mixture was also unsuccessful in the case of a mild nitration method with nitronium tetrafluoroborate as a nitration agent in sulfolane.[2]

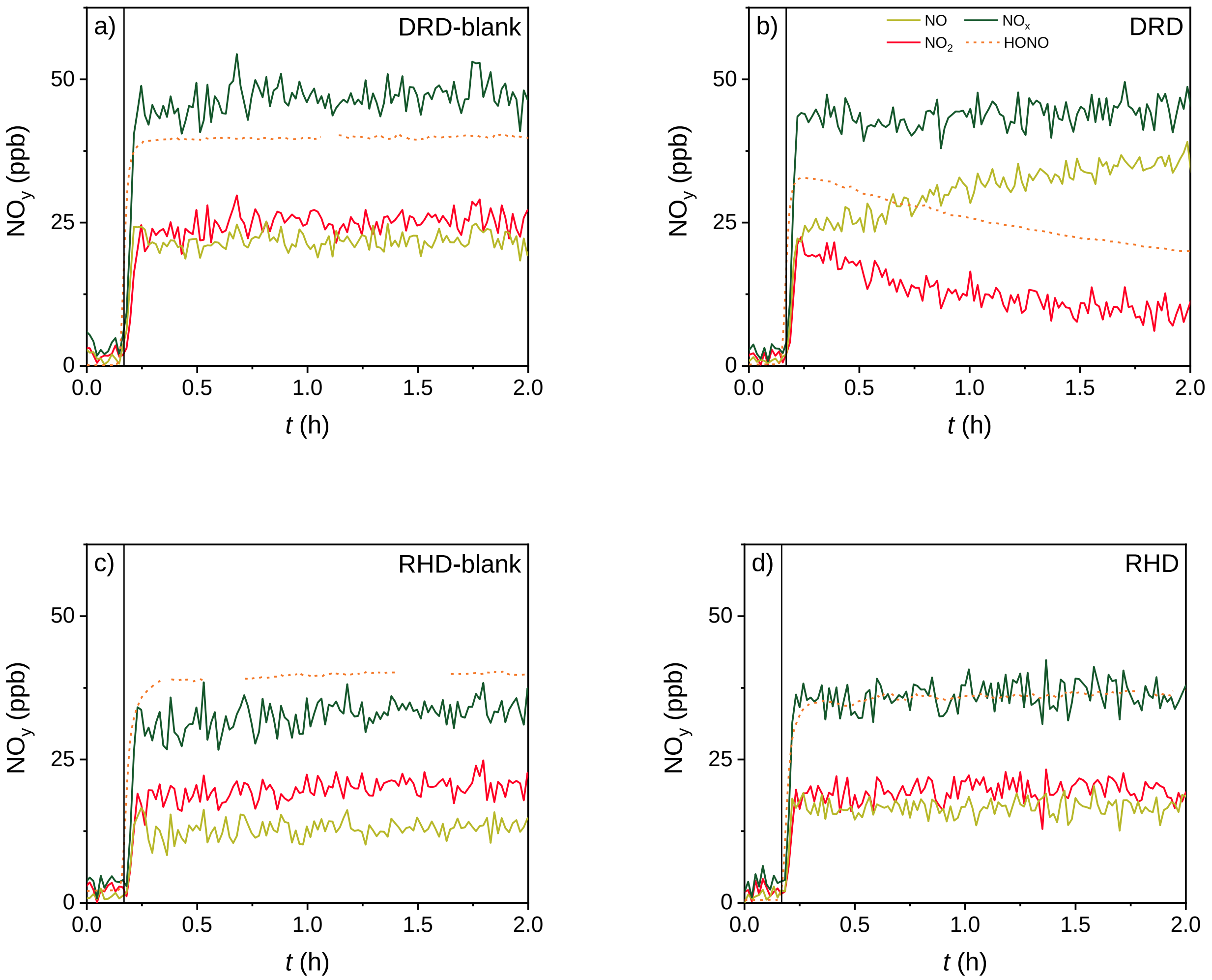

Figure S1. HONO concentration (LOPAP) and levels of NO, NO_2, NO_x ($NO+NO_2$) as measured by a photolytic converter (PLC). Experiments were performed in the dark: in dry air (DRD) **a)** without (blank) and **b)** with guaiacol, and at 80% RH (RHD) **c)** without (blank) and **d)** with guaiacol. Vertical lines denote seed injection in the chamber.

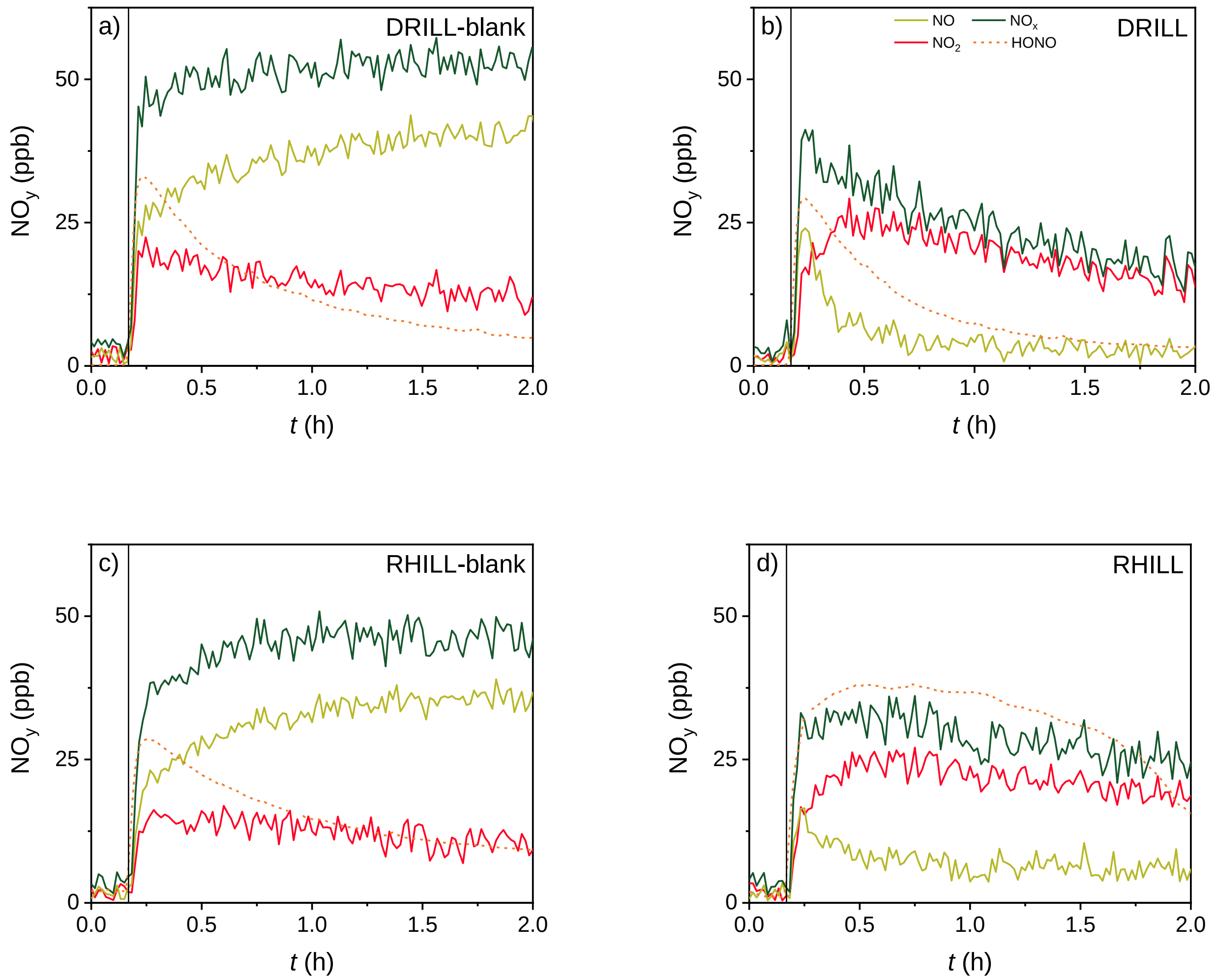

Figure S2. HONO concentration (HONO) and levels of NO, NO_2, NO_x ($NO+NO_2$) as measured by a photolytic converter (PLC). Experiments were performed under illumination: in dry air (DRILL) **a)** without guaiacol (blank) and **b)** with guaiacol, and at 80% RH (RHILL) **c)** without (blank) and **d)** with guaiacol. Vertical lines denote seed injection in the chamber.

Table S1. The list of products with retained aromatic ring as detected by ESI(−) LC-TOFMS. Identification was based on the mass of molecular ion (M^-) and the comparison with standard compounds. Gas-phase and particulate-phase concentrations (µg m^{-3}) and product yields (in % for those >0.1%) are also listed for all experimental conditions. Where no commercial standard was available, concentrations are estimated with use of a surrogate standard.

**Name**	**Formula**	**M^- *m/z* (t_r / min)**	**DRD**		**RHD**		**DRILL**		**RHILL**		**Product yield / %**
			**gas**	**part.**	**gas**	**part.**	**gas**	**part.**	**gas**	**part.**	
4-Nitrocatechol	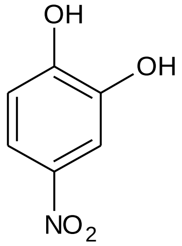	154 (20.3)	0.045[a]	0.25[a]	0.018[a]	0.015[a]	0.22	0.93	2.61	8.04	DRD: 0.1<br>RHD: <0.1<br>DRILL: 0.4<br>RHILL: 3.6
Hydroxy-nitroguaiacol[b,c]	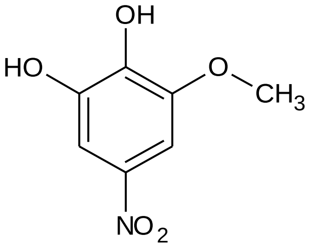	184 (23.0)	ND	ND	ND	ND	0.033	0.053	0.065	0.21	DRILL: <0.1<br>RHILL: <0.1
Nitrated pyrogallol and isomeric analogues[c,d]	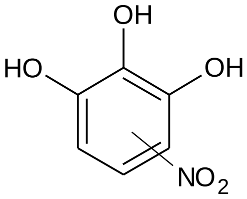	170 (13.2, 17.6, 19.5)	ND	ND	ND	ND	0.041, 0.028, 0.032	0.17, 0.073, 0.016	0.43, 0.039, 0.024	3.3, 0.025, 0.289	DRILL: 1.3<br>RHILL: 0.2

Continues on the next page…

Name	Formula	M^{-} *m/z* (t_r / min)	DRD		RHD		DRILL		RHILL		Product yield / %
			gas	part.	gas	part.	gas	part.	gas	part.	
4- Nitroguaiacol	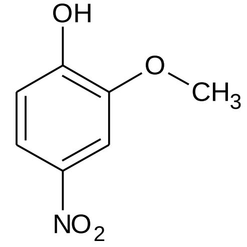	168 (26.8)	1.2	0.005	0.25	< LOQ	1.9	0.094	1.8	0.007	DRD: 0.4<br>RHD: <0.1<br>DRILL: 0.7<br>RHILL: 0.6
5-Nitroguaiacol	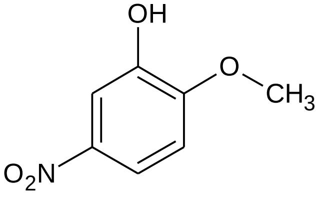	168 (26.3)	ND	ND	ND	ND	<0.001	0.004	0.013	0.080	DRILL: <0.1<br>RHILL: <0.1
6- Nitroguaiacol	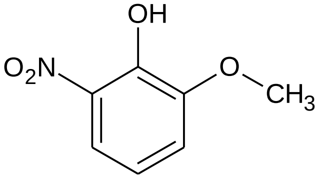	168 (27.7)	0.92	0.022	0.15	ND	0.047	0.089	1.2	1.3	DRD: 0.3<br>RHD: <0.1<br>DRILL: <0.1<br>RHILL: 0.8
4,6-Dinitroguaiacol	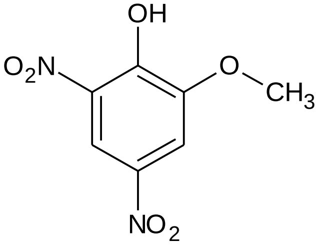	213 (28.7)	< LOQ	< LOQ	0.002	< LOQ	0.058	0.004	0.014	< LOQ	DRD: <0.1<br>RHD: <0.1<br>DRILL: <0.1<br>RHILL: <0.1

Continues on the next page…

Name	Formula	M^{-} *m/z* (t_r / min)	DRD		RHD		DRILL		RHILL		Product yield / %
			gas	part.	gas	part.	gas	part.	gas	part.	
3,5-Dinitrocatechol[c]	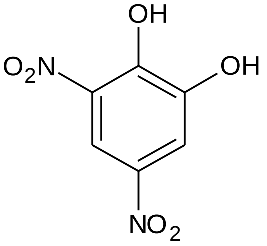	199 (27.9)	ND	ND	ND	ND	0.024	0.23	0.033	0.090	DRILL: <0.1<br>RHILL: <0.1

ND = not detected; Product yield is calculated as the ratio of the concentration of a particular product (gaseous+particulate) to the initial concentration of gaseous reactant (GUA); [a]likely originates from the impurity – an estimate of product yield is based on a postulate of 1% CAT as impurity; [b]tentative structure; [c]4-nitrocatechol standard was used for quantification; [d]three peaks with *m/z* 170 were observed, one is assigned as nitropyrogallol (identity was confirmed by the *de novo* synthesis from pyrogallol), the other two are likely its isomeric forms.

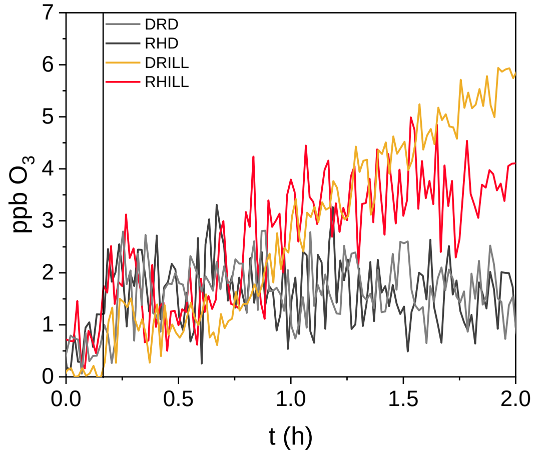

Figure S3. Ozone levels in the chamber at different experimental conditions: dry dark (DRD), humid dark (RHD), dry illuminated (DRILL), and humid illuminated (RHILL). Vertical lines denote start of experiment by seed injection.

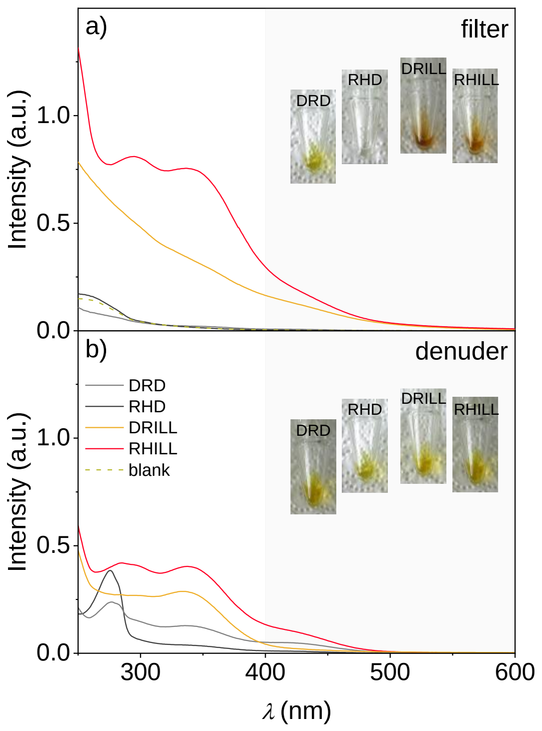

Figure S4. UV-Vis absorption spectra of a) filter and b) denuder extracts exhibiting characteristic absorption bands of identified phenolic compounds: 275 nm – guaiacol, 290 and 340 nm – nitrated phenols. Both samples correspond to the same air mass, whereas extraction recoveries were not assessed.

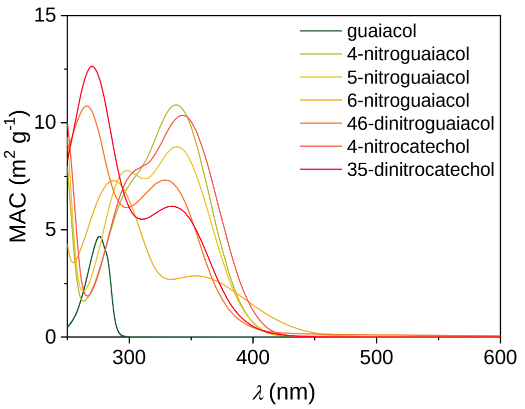

Figure S5. Absorption spectra of guaiacol and identified ring-retaining nitration products.

REFERENCES

1. Crivello, J. V., Nitrations and oxidations with inorganic nitrate salts in trifluoroacetic-anhydride. *J. Org. Chem.* **1981,** *46*, (15), 3056-3060.
2. Olah, G. A.; Narang, S. C.; Olah, J. A.; Lammertsma, K., Recent aspects of nitration - new preparative methods and mechanistic studies (a review). *Proc. Natl. Acad. Sci. U. S. A.* **1982,** *79*, (14), 4487-4494.
